# The American Medical Almanac for 1841

**Published:** 1841-07

**Authors:** 


Art. V.—*The American Medical Almanac for* 1841. By J. V. C. Smith, m.d. Vol. III. Continued annually.—*Boston*, 1841. 18mo, pp. 148.

This is a valuable little work, which we really envy America the possession of; as, since Mr. Farr discontinued his admirable Almanac, we have nothing of the same kind in England. Besides the usual contents of an almanac, it possesses all the requisites of a daily memorandum book, and contains many valuable statistical and medical documents. We have, for instance, a full account of the medical institutions of the United States and of Canada; the statistics of Insanity, and Institutions for the Insane, by Dr. Woodward; a manual of auscultation, under the modest title of "Short Sentences," by Dr. Bowditch; Statistics of the Massachusett's Hospital, by Dr. Turner; Statistics of Phrenology; an account of the operation for Strabismus, by Dr. Dix; an account of Dislocations, by Dr. Capen; of Dissection Wounds, by Dr. Lane; list of the Medical Journals, &c. &c. As a specimen of the materials composing this interesting little volume, we select the following, chiefly on account of its brevity:

"*Statistical Account of the most important Surgical Operations performed in Hartford County, Conn., for the last ten years preceding Jan. 1st,* 1840. With Remarks by A. Brigham, m.d., Superintendent of the Retreat for the Insane, Hartford, Ct.

"Hartford county, the northern border of which joins Massachusetts, and lies in latitude 42 deg. forms nearly a square, about 30 miles in length north and south, and 25 in width, and is intersected nearly in the middle by the Connecticut river.

"The soil is rich, various, and fertile, and for the most part highly cultivated. A great variety of manufactures are carried on in the county, employing in the cotton and woollen manufacture alone, in 1830, a capital of 600,000 dollars.

"There are 21 towns in the county, and numerous and pleasant villages. Population in 1830, 51,141; in 1840, 55,725. The following are the number of amputations and other important surgical operations which have been performed in this county during the last ten years; and I have no reason to believe they vary much from the number usually required in a population of 50,000. This account, therefore, may serve to undeceive and to benefit young physicians, who expect to obtain considerable income from surgical operations, and consequently neglect to qualify themselves so thoroughly as they should, to perform well the other and every-day duties of their profession, and upon which they must chiefly rely for support.

1. Amputations of the thigh, *ten;* for the following causes—*Five* for compound fractures with injury of joints; *three* for white swellings of the knee joint; *one* for fungus hæmatodes tumour of the leg; and *one* for mortification of the leg. Six recovered, two of the five cases of injury died, also the case fungus hæmatodes and that of mortification.

"2. Amputations of the leg, *four;*—*two* of them for accidents, *one* for frostbite, *one* for fungus hæmatodes. All but the last recovered.

"3. Amputations of arms, *eight*—five lower and three upper;—*six* for accidents, *two* for fungus hæmatodes; two of the accidental and one of the fungus hæmatodes died.

"4. Trephining, *five* cases; all but two died.

"5. Operations for strangulated hernia, *two;* both recovered. No other case has been heard of requiring an operation. Trusses are generally worn by those afflicted with hernia. Hull's most used.

"6. *Aneurism*—but one case requiring an operation. The popliteal artery was the one affected;—the femoral was tied and the man recovered.

"7. *Lithotomy.* No one so far as I have heard has required this operation in this county, for the last ten or even for the last thirty years. Cases of calculi are quite rare in Connecticut.

"8. There have been a few amputations of the breast of females; several operations for *hydrocele;* and a considerable number for the removal of enlarged *tonsils.*

"Though the county is a wealthy one, and comparatively but few poor people in it, yet the surgical operations alluded to have been mostly among the poor. Five of the ten persons who underwent amputation of the thigh were too poor to pay anything, and all but one of those who had the leg amputated. But two of the eight cases of amputated arm were able to pay, and only *one* of those trephined. The cases of hernia occurred in poor people, but the friends paid a little. The case of aneurism was in a town pauper. The whole sum realized from all the above mentioned amputations, trephinings, and operations for aneurisms and hernia is less than 350 dollars.

"There are 76 physicians in the county, most of whom obtain a good support from their professional business; but, as is very evident from the above account, they derive but little income from important surgical operations."

